# Shared Pathogenetic Features Between Common Variable Immunodeficiency and Sjögren’s Syndrome: Clues for a Personalized Medicine

**DOI:** 10.3389/fimmu.2021.703780

**Published:** 2021-07-12

**Authors:** Luca Quartuccio, Ginevra De Marchi, Simone Longhino, Valeria Manfrè, Maria Teresa Rizzo, Saviana Gandolfo, Alberto Tommasini, Salvatore De Vita, Robert Fox

**Affiliations:** ^1^ Rheumatology Clinic, ASU FC, Udine, Italy; ^2^ Department of Medicine, University of Udine, Udine, Italy; ^3^ Pediatric Immunology, IRCCS Burlo Garofolo, Trieste, Italy; ^4^ Department of Medical Sciences, University of Trieste, Trieste, Italy; ^5^ Rheumatology Clinic, Scripps Memorial Hospital and Research Foundation, La Jolla, CA, United States

**Keywords:** immunodeficiency, Sjögren’s syndrome, autoimmunity, lymphoproliferation, B cells

## Abstract

Common variable immunodeficiency disorders (CVID) are a group of rare diseases of the immune system and the most common symptomatic primary antibody deficiency in adults. The “variable” aspect of CVID refers to the approximately half of the patients who develop non-infective complications, mainly autoimmune features, in particular organ specific autoimmune diseases including thyroiditis, and cytopenias. Among these associated conditions, the incidence of lymphoma, including mucosal associated lymphoid tissue (MALT) type, is increased. Although these associated autoimmune disorders in CVID are generally attributed to Systemic Lupus Erythematosus (SLE), we propose that Sjogren’s syndrome (SS) is perhaps a better candidate for the associated disease. SS is an autoimmune disorder characterized by the lymphocytic infiltrates of lacrimal and salivary glands, leading to dryness of the eyes and mouth. Thus, it is a lymphocyte aggressive disorder, in contrast to SLE where pathology is generally attributed to auto-antibody and complement activation. Although systemic lupus erythematosus (SLE) shares these features with SS, a much higher frequency of MALT lymphoma distinguishes SS from SLE. Also, the higher frequency of germ line encoded paraproteins such as the monoclonal rheumatoid factor found in SS patients would be more consistent with the failure of B-cell VDJ switching found in CVID; and in contrast to the hypermutation that characterizes SLE autoantibodies. Thus, we suggest that SS may fit as a better “autoimmune” association with CVID. Examining the common underlying biologic mechanisms that promote lymphoid infiltration by dysregulated lymphocytes and lymphoma in CVID may provide new avenues for treatment in both the diseases. Since the diagnosis of SLE or rheumatoid arthritis is usually based on specific autoantibodies, the associated autoimmune features of CVID patients may not be recognized in the absence of autoantibodies.

## Introduction

Common variable immunodeficiency disorders (CVID) are a group of rare diseases of the immune system and the most common symptomatic primary antibody deficiency in adults. They comprise a group of disorders with similar antibody deficiency but a myriad of different aetiologies, most of which remain poorly understood (1–8). CVID are sometimes complicated with autoimmune features (9–11). Several biological mechanisms have been recently implicated in the development of these complications, including the decrease in the number of circulating switched memory B cells, CD21^low^ B cell expansion, interferon (IFN) signature and B-Cell Activating Factor (BAFF) hyper-expression, and they will be addressed in the subsequent paragraphs of this review. All of these mechanisms prevent the emergence of somatic mutation among the autoantibodies in CVID patients. Thus, CVID provides an opportunity to understand processes such as neutropenia, thrombocytopenia, and lymphoproliferation in the absence of the affinity selected autoantibodies that we normally invoke as pathogenetic mechanisms.

It is worth recalling the original studies by Kunkel et al. (12) pointed out that germ line genes (encoding both heavy and light chains) were found as autoantibodies in patients with Waldenstrom’s macroglobulinemia that had not undergone significant affinity selected maturation and recombination. For example, the germ line encoded antibodies with mixed cryoglobulin or cold agglutinin activity were sequenced and found to have a limited repertoire that was defined as conserved “idiotypes” and later found to have sequence due to germ line encoded heavy and light chains. Of interest, similar limited expression of light chains was found in the rheumatoid factor (RF) of Sjögren’s syndrome (SS) patients (i.e., the 17-109 idiotype) but not in the highly variable light chains of RF in Systemic Lupus Erythematosus (SLE) patients (13). Further, the B-cell lymphomas of SS show a marked limitation of their surface immunoglobulin heavy and light chains (14). In contrast, autoantibodies with extensive somatic diversification mechanisms are the hallmark of SLE and these patients do not have the elevated frequency of B-cell Mucosa Associated Lymphoid Tissue (MALT) lymphomas (15).

In this review we look at the potential link between CVID and SS based on the high frequency of lymphoma in both groups. This contrasts with the most reviews that suggest SLE is the main “associated” systemic autoimmune disorder. This change of view is more than semantic and emphasizes that SS is a disorder of “aggressive” hyperactivated lymphocytes that infiltrate tissues in comparison to SLE that is characterized by its pathogenic antibodies that play a role through immune complexes and complement activation.

## Clinical Features, Autoimmune Aspects and Heterogeneity of CVID

CVID represents the most frequent clinically expressed primary immunodeficiency (PID) in adults, accounting for more than 50% of cases of PIDs (1, 2). Worldwide geographic differences in prevalence are the consequence of discrepancies in diagnostic methods, disease awareness and data registration (3).

The term CVID was firstly coined in 1971 by the World Health Organization to express a diagnosis of exclusion from other antibody deficiency syndromes with more specific clinical and inheritance patterns (4). Since then, CVID diagnostic criteria have been revised many times (1, 5–7), matching the evolution in the clinical, immunological and genetic knowledge on the disease (7, 8).

In 2008, Chapel et al. firstly categorized CVID complications, identifying five distinct phenotypes: no complications, autoimmunity, polyclonal lymphocytic infiltration, enteropathy and lymphoid malignancy (9). Subsequently, other studies attested the classification of CVID based on the presence of complications, and the concomitance of certain features, as autoimmunity, lymphocytic interstitial lung disease and lymphoid hyperplasia, was noted (10, 11).

More recently, the 2016 International Consensus document on CVID supported further analysis on the associations between genetics, clinical presentation, disease severity and immunotype, allowing the distinction into “infection-predominant”, “inflammatory predominant” and “autoimmunity predominant” entities (1).

The latest European society of Immune Deficiency (ESID) (2019) diagnostic criteria include autoimmune and inflammatory conditions as primary clinical presentations, in addition to laboratory abnormalities (8).

In fact, it has emerged that at least 30% of patients show additional non-infectious conditions, as autoimmune, autoinflammatory, granulomatous, lymphoproliferative and/or malignant complications, especially in patients with low fraction of isotype switched memory B cells (1, 6, 11, 16).

Autoimmune diseases can be observed before CVID diagnosis in up to 17.4% of patients and as the only clinical manifestation at the time of diagnosis of CVID in 2.3% of patients (17).

Autoimmune and autoinflammatory conditions reported in CVID are summarized in **Table 1**.

**Table 1:** Autoimmune and autoinflammatory conditions reported in CVID.

Classification	Specific disorder
**Autoimmune cytopenias**	Immune Thrombocytopenic Purpura Autoimmune Haemolytic Anemia Autoimmune Neutropenia
**Organ-specific disorders**	
***Lung***	Lymphoid Interstitial Lung Disease Granulomatous Lymphocytic Interstitial Lung Disease
***Gastrointestinal tract***	Autoimmune Enteropathy Inflammatory Bowel Disease Celiac Disease Pernicious Anemia
***Endocrine system***	Thyroiditis Type 1 Diabetes
***Skin***	Psoriasis Vitiligo
**Rheumatic diseases**	Inflammatory Arthritis Sjögren’s Syndrome Systemic Lupus Erythematosus Vasculitis Behçet’s Disease

Systemic autoimmune diseases, properly rheumatic diseases, were found in 5.9% of all cases in a cohort of 870 CVID patients analyzed from the USIDNET registry, accounting for almost 40% of the detected global autoimmune manifestations. One third of patients with CVID-associated rheumatologic disorders had an additional inflammatory complication or malignancy (18). Among CVID patients affected by rheumatologic conditions, a female predominance has been noted, while inflammatory arthritis has been reported as the most frequent rheumatological manifestation (3%), followed by SS (11/870 in USIDNET registry), SLE, vasculitis and Behçet’s disease and others (18–20).

Importantly, in CVID patients, the overall risk of lymphoid malignancies [e.g. extra-nodal B cell non-Hodgkin lymphoma (NHL)] ranges between 2 and 10% (11, 20), while the risk of gastric cancer was reported as 10-fold increased (21).

## CVID and Autoimmunity

The pathogenesis of autoimmune complications in CVID is poorly understood, as well, and is counterintuitive because these patients are defined by their inability to make antibodies yet still mount autoimmune reactions. Some general assumptions may support this paradox:

a) the co-existence of hypo- and hyper-immune states in the same individual at the same point in time is not implausible given the complexity of the immune system;
b) both T and B cells abnormalities may contribute to the development of autoimmunity in CVID patients;
c) increased autoreactive B cells and reduced T regulatory cells may be involved in the pathogenesis of autoimmunity in CVID.

Studies on B and T cell immune dysregulation found many possible responsible factors for autoimmunity appearance, such as the expansion of CD21^low/-^ B cells and related reduction of T regulatory cells (22); the reduction of switched memory B cells (23, 24); the low levels of naïve CD8+ (25) and CD4+ (22, 25, 26) T cells and the elevated T helper 1 and IFN gamma signature (27), related to the increase in T helper 1 and follicular T CD4+ cells (28, 29). Conflicting evidence emerged on the role of BAFF and IL-7 (10, 30–32).

### Challenge of Identifying SS in CVID

Even if autoimmune clinical manifestations reported in CVID mostly resemble SLE (autoimmune cytopenias in particular), we suggest that SS may fit as a better “autoimmune” association.

SS diagnosis is not as simple as you’d think. One recent study from academic institutions with expertise in SS has shown that almost 50% of patients diagnosed as SLE with dry eye symptoms actually had SS when the patients were re-examined for the presence of anti-SSA antibody and other clinical features of SS (33). Moreover, after the patient is initially labelled as SLE, it is rare that the underlying diagnosis is re-examined. As a result, SS patients with extraglandular manifestations, that might benefit from new trials of therapy, could be never considered.

Moreover, patients affected by CVID might show SS typical manifestations even in the absence of SS related autoantibodies, determining a condition resembling seronegative SS. These clinical manifestations include both glandular (e.g. sicca symptoms) and extraglandular manifestations (e.g. constitutional manifestations, interstitial lung disease, tubular nephritis, haemolytic anemia, thrombocytopenia) (34). Thus, patients with CVID should be investigated more thoroughly for SS-related symptoms and studied in depth with functional, instrumental and histopathological tests (e.g. minor salivary gland biopsy) in addiction to laboratory parameters.

### SS-Like Features in CVID Patients Who Lack Autoantibody to SS-A

The characteristic pathologic picture in both glandular and extraglandular manifestations of SS is the “aggressive” lymphocyte that infiltrates tissues. This may be reflected in the “focus score” that counts clusters of lymphocytes in a minor salivary gland biopsy, the analogous infiltrates of the lacrimal glands, the lymphocytic clusters in the lung in lymphocytic interstitial pneumonitis (LIP) or the markedly increased frequency of lymphoma.

Although elegant models have shown that SS-A is a chaperone molecule to both single and double stranded viral nucleotides, it is the resistance of SS-A to breakdown in the apoptotic bleb that makes it an attractive candidate for perpetuating the autoimmune cycle. The binding to antibody to SS-A (whose production is closely linked to HLA-DR3) provides a mechanism for Fc internalization of the SS-A/hYRNA complex with subsequent internalization and translocation to the toll-like receptor (TLR) (35). Yet, the finding of lymphoid infiltrates and lymphoma and CVID indicate that there is more to the story.

In fact, since CVID patients lack detectable circulating autoantibodies including anti-SS-A estimation of the role of SS pathogenetic factors in CVID is likely to be grossly underestimated. For example, it has been shown that activation of TLR receptors by viral and bacterial nucleic acids plays a role in CVID by promoting IFN alpha pathway rather than TNF alpha upon stimulation (36). Also, non-coding small RNAs are important (37). Other common factors such as T-follicular type and T-helper type phenotype and B-cells expressing low levels of cellular surface CD as well as reciprocal decrease in regulatory T-cells and isotype switched memory B cells will be reviewed below.

Thus, the lesson for rheumatologists from CVID is that we have considered the cardinal feature of SS as SS-A antigen and antibody that targets. However, also in the absence of antibody to SS-A we see the lymphocyte aggressive features that characterize its dysautonomic features (dry eyes, dry mouth, dry skin, interstitial pneumonitis, interstitial nephritis, and increased frequency of lymphoma).

## Relevant Clues From the Genetics of CVID

While CVID is mainly a polygenic and multifactorial disease, recent technical advances in next generation sequencing (NSG) allowed to discover a monogenic cause in up to 15-30% of cases (38, 39). Thirteen monogenic mutations associated with CVID are listed on the Online Mendelian Inheritance in Man (OMIM) database. Among them, some are specifically associated with autoimmunity, and are listed in **Table 2**.

**Table 2:** Mutations associated with CVID and autoimmunity.

Gene	Effect	Result on immune system
**TNFRSF13B**	Loss of function in TACI	Breakdown of B cell tolerance
**ICOS**	NF-κB deficiency	Breakdown of B cell tolerance
**NFKB1/NFKB2**	NF-κB deficiency	Breakdown of B cell tolerance
**STAT3**	Gain of function	Th17 cell expansion
**LRBA**	Loss of function	T cell activation
**CTLA4**	Loss of function	T cell activation
**PIK3CD**	Gain of function	Impaired T-cell and B-cell development and function

TNFRSF13B, TNF Receptor Superfamily Member 13B; TACI, Transmembrane activator and CAML interactor; ICOS, Inducible T Cell Costimulator; nuclear factor of kappa light polypeptide gene enhancer in B-cells 1; nuclear factor of kappa light polypeptide gene enhancer in B-cells 2; STAT3, Signal Transducer and Activator of Transcription 3; LRBA, LPS responsive beige-like anchor protein; CTLA4, Cytotoxic T-Lymphocyte Antigen 4; PIK3CD, phosphatidylinositol-4,5-bisphosphate 3-kinase catalytic subunit delta.

TACI may be involved in the central B cell tolerance and that reduced function results in the loss of tolerance and resultant autoimmunity (40).

ICOS and NF-κB deficiencies lead to CVID-like immunodeficiency syndromes and autoimmunity (37–39). Interestingly, dysregulation of NF-kB in glandular epithelial cells results in SS-like features (41), as well as expression of NF-κB at both the mRNA and protein level was up-regulated in SS-lymphoma-BAFF-RHis159Tyr-derived B cells, linking the innate to the adaptive immunity upregulation and lymphoma in SS (42).

STAT3 is thought to lead to autoimmunity by promoting the activation and expansion of autoimmunity-associated TH17 cells, a subtype of T cell deeply involved in the early mechanisms of autoimmunity (43–45).

LRBA and CTLA-4 are inhibitors of T cell, and their deficiencies cause excessive T cell activation and breakdown of immune tolerance, resulting in autoimmunity, as emerged even under checkpoint inhibitor therapy, namely ipilimumab (46–48).

Activated PI3Kδ syndrome (APDS) is characterized by impaired T- and B-cell development and (APDS) function, autoimmunity, and lymphoproliferation (49).

## Common Features Between CVID and Sjögren’s Syndrome

### Lymphoproliferation

Both CVID and primary SS are strongly related to lymphoproliferation and lymphoma, in particular B cell NHL and MALT-type lymphoma.

#### CVID and Lymphoproliferation

Data collected from 1091 CVID patients, showed that CVID patients with a lymphoproliferative pattern have a 2.5-fold increased risk of developing lymphoma. The most common forms of benign lymphoid hyperplasia in CVID are splenomegaly and lymphadenopathy, but lymphoid hyperplasia and polyclonal lymphoproliferative infiltrations frequently affect other organs and tissues such as lungs and gastrointestinal. The lung represents the prevalent extranodal site for lymphoproliferative disorders, which include follicular bronchiolitis, lymphoid interstitial pneumonia, and pulmonary nodular lymphoid hyperplasia (50). Chapel et al. confirmed polyclonal lymphoid infiltrate as a predictor of lymphoma in CVID, which increased by 5 times the risk of developing lymphoma (9). The main histotypes of lymphoma in CVID are represented by mature B-cell malignancies followed by Hodgkin’s lymphoma and rarely by MALT-type lymphomas (51–53).

The presence of a 2-step transformation mechanism is hypothesized, as in non-Hodgkin’s lymphoma (9). A benign lymphoproliferation is deeply linked to the immune dysregulation intrinsic to CVID patients, as observed in other primitive immunodeficiencies such as CTLA-4 haploinsufficiency and STAT 3 gain of function mutations and as do autoimmune diseases such as primary SS (43, 46, 54). In CVID, mutation of TACI, reduction of isotype-switched memory B cells, expansion of CD21^low/-^ B cells, expression of an IFN signature, expansion of inflammatory innate lymphoid cells and retained B cell function are all linked with development of autoimmunity and lymphoproliferation (19).

#### SS and Lymphoproliferation

B-cell clonal expansion is a key feature of SS and progression to B-cell lymphoma occurs in about 5% of patients. The progression from polyclonal, to benign clonal lymphoproliferation, to overt lymphoma in SS is one of the few human models in which one can study B-cell lymphomagenesis and its link to immune dysregulation (55).

The pathological hallmark of SS is MALT arising in chronically inflamed tissues, mainly in salivary glands, where inflammation, autoimmunity and lymphoproliferation coexist, creating a complex biological and immunological substratum that fuels autoreactive B lymphocytes persistence and promotes their proliferation, towards a clonal selection and a possible lymphoma development (54, 56).

In SS the prevalent histological type of lymphoma is marginal zone and particularly MALT lymphoma of the salivary glands but other histotypes are described and lung is one of the prevalent organ targets of lymphoproliferation besides exocrine glands (57).

In primary SS, splenomegaly and lymphadenopathy also represent well established lymphoma risk factors, in addition to salivary gland swelling (58, 59). Moreover, in primary SS patients the typical histopathological feature of ILD is LIP (60, 61). Both lung and stomach are other sites of lymphoma development in primary SS, other than salivary and lacrimal glands (58, 59).

### BAFF Hyperexpression

#### CVID and BAFF Hyperexpression

Knight et al. demonstrated high serum levels of BAFF, APRIL and TACI in CVID patients, however, they didn’t find a correlation with immunological or clinical phenotypes (30).

Similarly, Kreuzaler et al. showed increased BAFF serum concentration in CVID patients, without a clear correlation with clinical parameters, immunodeficiency-related inflammatory disease and B cell subsets (62).

On the contrary, Maglione et al. showed that ILD progression in CVID correlates with increased levels of IgM, particularly with the production of IgM within B cell follicles in lung parenchyma; the main stimulator of pulmonary B cell hyperplasia seems to be BAFF, which was increased both in the blood and in the lung of CVID-ILD patients (63).

#### SS and BAFF Hyperexpression

Among the systemic autoimmune diseases, SS showed the highest serum levels of BAFF (64–69). In mice model, the overexpression of BAFF in mice leads to hyperplasia, autoimmunity, hyperglobulinemia and splenomegaly, while the normal expression of BAFF allows B cell survival and maturation (70). In BAFF transgenic mice there’s an excessive survival signal to autoreactive B lymphocytes, probably linked to a dysregulation of tolerance at the splenic level, where they observed an enlargement of marginal zone B-cell subset. B cells with an Marginal Zone (MZ)-like phenotype infiltrate the salivary glands of BAFF transgenic mice. Parallelly, unbalanced BAFF production in the lymphoid infiltrates of the salivary glands of primary SS patients promote recruitment of a specific and potentially pathogenic subpopulation of B cells (71). Epithelial cells also produce BAFF, thus supporting the hypothesis of the crucial role of BAFF in the pathogenesis of primary SS, by an immune dysregulation through an autocrine pattern of self-stimulation (72). High levels of BAFF were correlated with the specific autoantibodies of SS, anti-SSA/SSB, and BAFF was also found mainly in local lymphoid and inflammatory microenvironments (73). In addition, BAFF upregulation correlates both with primary SS disease activity and B cell prelymphomatous and malignant lymphoproliferative disorders (74). Genetic mutations in BAFF-mediated pathway may significantly contribute to this risk of malignant evolution (42). The efficacy of belimumab, a human monoclonal antibody targeting soluble BAFF, approved for the treatment of SLE, in a phase II clinical trial of primary SS strongly supported the pathogenic role of BAFF in this autoimmune disease (75, 76), and a phase III trials of belimumab in co-administration with rituximab in primary SS is ongoing (NCT02631538).

#### B Cell Abnormalities

Two B cell subpopulations have been shown to play a central role in both these entities: switched memory B cells and CD21^low/-^ B cells. The formers are CD19+CD27+IgM-IgD- memory B cells which have undergone the isotypic switch; the latter are a peculiar B cell subset that under expresses CD21, a coreceptor of BCR, and at the same time expresses higher levels of IgM, CD11c, CD19 and CD95. CD21^low/-^ B cells belong to a unique anergic B cell population which is polyclonal, pre-activated, enriched in autoreactive clones and can express highly autoreactive antibodies, including antinuclear antibody and RF (77, 78). The nature of this cell population was extensively studied also in the context of HCV related cryoglobulinemia; in both HCV related cryoglobulinemia and CVID, an anergic subset of CD21^low/-^ B cells appears expanded and characterized by high constitutive expression of extracellular signal regulated kinase (pERK) (79–81). Moreover a BAFF hyperexpression and aberrant type I and II IFN response are thought to support CD21^low/-^ B cell population, suggesting a profound interconnection between dysregulated innate and adaptative immunity (77, 82).

#### CVID and B-Cell Abnormalities

Since the early 2000s the role of switched memory B cells and CD21^low/-^ B cells in CVID has been investigated. Warnatz et al. observed a significant decrease in class-switched B memory cells in CVID patients compared to healthy controls and identified a CVID subgroup, clinically characterized by splenomegaly and autoimmune disorders, with a high proportion of CD21^low/-^ B cells. These findings suggested a correlation between low switched memory B cells, increased CD21^low/-^ B cells and autoimmune and lymphoproliferative disorders in CVID (23, 83). In particular, the reduction of IgM-, IgD- CD27+ switched memory B cells represents the most common aberration in CVID, and it correlates with decrease in serum IgA and IgG levels. Sanchez Ramon et al. found that levels of switched memory B cells <0.55% had 3.3-fold higher risk to correlate with autoimmune disease (84). Many other studies confirmed these results (19, 77, 78, 85). Of note, in the large cohort of USIDNET register lower levels of switched B memory cells were observed in CVID-Rheum group (18).

#### SS and B-Cell Abnormalities

On the other hand, there is strong evidence of unbalance of B cell subpopulations also in SS. Many authors found that memory and switched memory B cells are reduced in primary SS compared to controls (86–88), and this unbalance appears to be related to disease duration and activity (88). Saadoun et al. found an increase of CD21^low/-^ B cells in primary SS and in particular in primary SS with lymphoproliferative disorders, suggesting a key role of this B cell population in SS related lymphomagenesis (89, 90). As in CVID, they found that CD21^low/-^ B cells are enriched in autoreactive clones and express highly autoreactive antibodies, such as RF, as a consequence of a chronic antigenic stimulation; this mechanism was preliminary linked to lymphoproliferation in primary SS and in HCV infection (91). The persistence of these cells can represent the initial reservoir for monoclonal expansion of a transformed clone and drive to B cell lymphoproliferation (90). Other papers support the correlation between the presence of B cell NHL in primary SS patients and the proportion of circulating CD21^low/-^ B cells (92, 93).

Rituximab can efficiently target those subtype of B cells in CVID with autoimmune or nonmalignant lymphoproliferative manifestations, as well as other therapeutic approach aiming to specifically deplete CD21^low/-^ B cells through an anti FcRL5 recombinant immunotoxin, originally employed in cryoglobulinemic vasculitis (94).

### Interferon Signature

Type I and II IFNs are cytokines which play a central role in regulation of immunity and inflammation. Since their contribution in loss of immunotolerance, they were considered as potential therapeutic targets of drugs such as anifrolumab, an anti-type I IFN receptor monoclonal antibody [NCT 02446899], in SLE. Type II IFN (IFN gamma), instead, appears more significant in diseases characterized by a prominent lymphoproliferative component, such as primary SS (95).

This increased expression of canonical IFN stimulated genes in tissues and in circulating blood cells is defined IFN signature, which is one of the possible key items shared among primary SS and CVID with autoimmunity. In particular, an upregulated IFN signature expression distinguishes CVID patients with inflammatory complication, including autoimmunity and, at the same time, it is a hallmark of various systemic autoimmune diseases such as SLE, systemic sclerosis, myositis and primary SS (19, 96).

#### CVID and Interferon Signature

Regarding CVID, few papers have been published on the role of IFN signature (27, 97).

Subjects with CVID and inflammatory/autoimmune conditions displayed significantly over-expressed IFN-related transcriptional modules and pronounced downregulation of transcript related to the B cell, plasma cell and T cell modules as compared to CVID without these conditions or controls (27). Also, a significant expansion of circulating IFN gamma producing innate lymphoid cells (typically ILC3) in CVID patients with noninfectious complications compared to those without and identified these cells in the affected mucosal tissues of lung and gastrointestinal tract (97). Notably, these cells, that also correlate with inflammation and produce IL17, were detected in salivary glands of primary SS patients, although their role in this autoimmune disease is not known (98). Unger et al. demonstrated a Th1 skewed CD4 T-cell population that highly express IFN-gamma both in peripheral blood and in lymph-nodes. Notably, in the same study, IFN gamma immune environment is thought to participate in expansion of circulating CD21^low/-^ B cells (29).

#### SS and Interferon Signature

On the contrary, the role of IFN signature in SS has been highlighted by many studies (99, 100). The presence of IFN-induced gene expression was demonstrated in salivary glands, peripheral blood mononuclear cells, isolated monocytes and B cells of primary SS patients and type I IFN signature was associated with higher disease activity and higher levels of autoantibodies (101–106). Type II IFN signature was also detected in salivary glands of primary SS patients (107). Two studies by Bodewes et al. confirmed the central role of overactivated innate immunity and IFN system in primary SS, particularly type II IFN (108, 109).

An aberrant activation of type I IFN response could drive autoantibody production, partly by direct activation of autoreactive B cells and partly by cytotoxic effect, accumulation of cellular debris and expression of autoantigen Ro52 (110, 111). Additionally, type I IFNs induce the expression of BAFF (109).

Interestingly, a prominent type I IFN signature was also associated with markers of B cell overactivity, such as anti-SSA antibodies, that can be attributed to type I IFN induced BAFF overproduction (106, 112). In the setting of lymphomagenesis both type I and II IFN transcript levels were considerably increased in minor salivary gland tissues from primary SS derived lymphoma, implying a direct role of these cytokines, and in particular IFN gamma, in this process (95).

Even if the main site of B cell hyperplasia and lymphoma development in CVID is the lung, also salivary glands can be involved in some cases (50–53, 113). Data suggest that IFN gamma could upregulate BAFF both in peripheral blood and in lung tissue, and locally BAFF could promote B cell survival and proliferation (63, 114).

## Use of “Anti-Rheumatic Therapies” in CVID and SS

Whereas immunoglobulin replacement therapy and improved anti-microbial drugs have significantly ameliorated CVID patients survival by reducing infectious complications (16), patients with CVID affected by at least one non-infectious complication still have significant higher risk of mortality compared to the other CVID patients, since these clinical manifestations do not respond to the antibiotic and immunoglobulin replacement therapy alone (11, 20). Thus, it appears that noninfectious complications, especially gastrointestinal and pulmonary involvement, constitute the most difficult aspects of the CVID patient management (1, 20, 115–117).

Over the last 5-10 years, rituximab has been used in various non-infectious CVID complications, such as autoimmune cytopenias, granulomatous lymphocytic interstitial lung disease (GLILD) and non-malignant lymphoproliferative syndromes (118). Of note, rituximab and, more recently, belimumab, as B-cell targeted therapies, have been applied in primary SS, and they resulted effective in particular in patients with systemic features (75, 76, 119). Combination strategies with both drugs are currently under evaluation in primary SS and also in other autoimmune diseases (69).

Also, abatacept, a CTLA-4 immunoglobulin fusion protein, showed good results as a replacement therapy in patients affected by CTLA-4 and LRBA deficiency (47, 120). In addition, tocilizumab and inhibitors of Janus Kinases (JAKs) were successfully trialed in patients with STAT3 gain of function mutations, as its activation occurs downstream of both IL-6 and JAKs (43, 121).

Yet, both tocilizumab and abatacept were employed as possible new treatments of primary SS, and even JAKs inhibitors are under evaluation in primary SS (NCT04496960).

The multicenter double-blind randomized placebo-controlled trial with tocilizumab in primary SS did not improve systemic features over 24 weeks of treatment compared with placebo (122); however, tocilizumab might be effective in contrasting SS-related articular and pulmonary involvement, such as refractory organizing pneumonia (123); moreover, tocilizumab has been recently approved by FDA for pulmonary fibrosis in systemic sclerosis (124).

Two open studies have assessed abatacept in primary SS; the first demonstrated the reduction in glandular inflammation and an increase in saliva production (125), while the second one showed the decrease of ESSDAI, ESSPRI, RF, and IgG levels but salivary and glandular functions did not improved (126). Finally, the phase III trial failed to demonstrate any clinical benefit of abatacept in primary SS (127).

On the other hand, leniolisib (CDZ173), a potent and selective oral inhibitor of PI3Kdelta (128), has been successfully used in a series of patients with APDS, in which PI3Kdelta gain-of-function mutation results in lymphoproliferation of the MALT, T-cell senescence and immunodeficiency. Leniolisib normalized B cells in APDS, and improved lymphoproliferation (129). A phase II clinical trial [NCT02775916] assessing the safety, pharmacokinetics, and preliminary efficacy of leniolisib in SS has been completed in January 2021, and preliminary data were presented in 2018 (130).

## Lessons From PID: Autoimmunity Is Not Just an Autoantibody!

First, SS may be probably underestimated in CVID, due to the absence of specific autoantibody, while specific symptoms may be unnoticed by specialists other than rheumatologists. On the other hand, patients with autoimmune diseases undergoing recurrent infections, or with peculiar features such as early onset or overlap syndrome should be evaluated for PID.

Second, the link between impaired B cell development, autoimmunity and lymphoma should be better elucidated based on the new growing knowledge in CVID.

Some CVID show B cell survival defects blocking the progression from transitional to naïve mature B cells, other CVID are characterized by class-switch recombination defects, impairing the evolution from follicular B cell to switched memory B cells, finally still other CVID display maturation defects into plasma cells (131). Nevertheless, the B cells in CVID are still able to produce autoantibodies, but they cannot isotype switch or affinity mature in response to new antigen challenge.

In X-linked agammaglobulinemia (XLA), BCR transcripts from peripheral blood CD19+ CD10+ IgM+ CD27−, emigrant mature naive B‐cells, represent the majority of peripheral blood B‐cells in XLA patients, and a higher proportion of which are self‐reactive and polyreactive antibodies and preferentially used VH1‐3 and VH4‐34 genes (132). Usually, the IgMkappa type encoded by the V(H)4-34 gene segment is the monoclonal immunoglobulin detected in primary chronic cold agglutinin disease (CAD), that is an autoimmune haemolytic anaemia induced by cold reactive autoantibodies (cold agglutinins) against erythrocyte surface antigens (133). Of note, unmutated VH4‐34+ B‐cells can be detected in patients with SLE memory compartment and VH4‐34‐expressing plasma cells appear to be clonally expanded during flares (134).

Interestingly, in ocular adnexal marginal zone B-cell lymphomas, which are observed also in SS, a strongly biased usage of V(H)4-34 in chlamydia negative patients was documented, suggesting the involvement of a particular stimulatory (auto-) antigen in their development (135). Similarly, the VH4 family usage of immunoglobulin gene rearrangement characterized also the MALT lymphomas of thyroid (136).

Yet, in Wiskott–Aldrich syndrome (WAS), a rare X‐linked PID with the classical clinical features of susceptibility to infections and autoimmune thrombocytopenia, memory B‐cells showed a low frequency of somatic hypermutation, while an increased usage of uncommon VH genes in transitional, naive and CD19^high^CD21^low^ B‐cells if compared with healthy controls, some of which are enriched in autoantibodies (VH4‐34 and VH4‐61) (137). Therefore, autoimmunity in the absence of B-cell switching is possible; when B-cells are frustrated in their normal path, they can proliferate in other pathways and use germ line genes that promote autoreactivity.

Importantly, in SLE undergoing rituximab, it was reported that B-cell abnormalities resolved after effective B cell depletion and immune reconstitution, including the frequency of autoreactive VH4.34 memory B cells (138), thus, possibly explaining the efficacy of rituximab in some autoimmune manifestations of PID.

In the same way, also anti-neutrophil cytoplasmic antibodies, that may play a pathogenic role in vasculitis, showed the use of germ-line VH4 family genes (139), as well as IgG anti-platelet autoantibodies in chronic immune thrombocytopenic purpura (140).

In CVID, the available BCR repertoire data are in line with the heterogeneity of the disease and generally show lower levels of somatic hypermutation, decreased repertoire diversity and longer CDR3 segments (141). The genetic heterogeneity of CVID made it difficult to better study the sequence encoded in their B cells.

Third, lymphoproliferation over autoimmunity is the hallmark of SS rather than SLE or other systemic autoimmune diseases. Indeed, SS more than SLE shows a high frequency of monoclonal gammopathy, with germ-line gene sequences recorded in lymphomas and Waldenström’s macroglobulinemia (i.e., 17-109 crossreactive idiotype) (142). Moreover, CAD represents a spectrum of clonal lymphoproliferative disorders overlapping with Waldenström’s macroglobulinemia itself (143).

In SS, a germline and coding polymorphism of TNFAIP3 (A20), a central gatekeeper of NF-kB activation, was found associated with lymphoma, linking the impaired control of NF-kB activation in B cells to autoimmunity and to the risk of lymphoma (144).

This concept has been recently reinforced particularly in mixed cryoglobulinemia secondary to SS, where the original Waldenström RF idiotypes were shown to be used in the monoclonal RF of mixed cryoglobulinemia in SS. Interestingly, in SS, at the beginning of the cascade of the events that lead to lymphoma and cryoglobulinemia, the unmutated V(D)J germ-line combinations with the characteristics of RF antibodies of Wa and Po public idiotypes, have very weak binding to self-IgG and this low affinity for IgG autoantigen would enable these newly formed B cells to evade central and peripheral B cell tolerance checkpoints and be activated transiently by IgG forming complexes with foreign antigens. The subsequent events are affinity maturation, somatic mutation acquisition and finally soluble accumulation of particular V(D)J mutations that compromise solubility of autoantibody-antigen complexes (145). It can be argued that in CVID, the first step described above is dramatically increased by mutations that promote breakdown tolerance and infections that facilitate proliferation of the expansion of autoreactive clones, leading to autoimmunity. In this regard, in SS, circulating IgG complexed with Ro and La ribonucleoproteins represents the driving force that induces low-affinity RF B cells to proliferate. Rare immunoglobulin mutations that improve affinity for self-IgG selectively allow some clones to emerge as dominant. In CVID, the levels of autoantibodies may be rarely found in circulation because their serum levels are extremely low, as also regularly occurred in idiopathic thrombocytopenic purpura or in chronic autoimmune neutropenia, since the neutropenia and thrombocytopenia may be due either to IgG Fc-mediated clearance in the spleen or due to destruction in periphery by routine clearance. Similarly, the very low amount of IgG anti-SS-A or SS-B, as well RF, may be cleared from the circulation or the low production may be confined in the target tissue (146). Deeper molecular and tissue studies in CVID with associated SS could support this hypothesis.

## Conclusions

**Figure 1:**
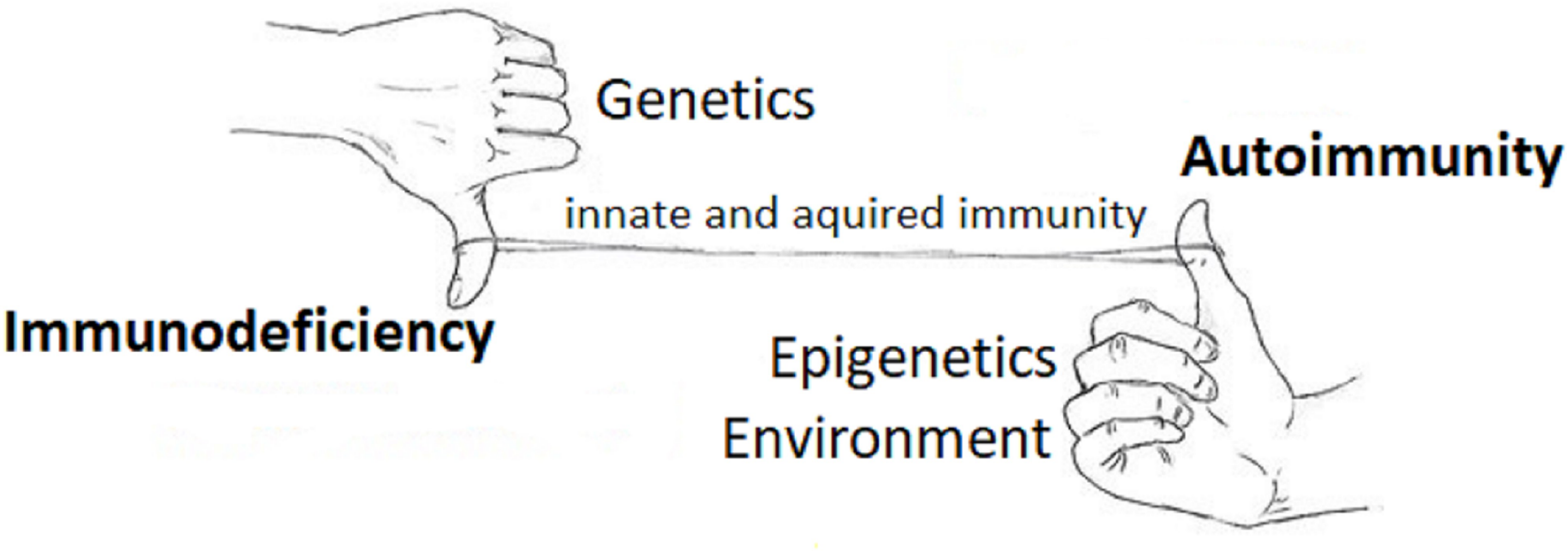
An integrated view of the immune system in primary immunodeficiency and autoimmune disorders. The figure illustrates the concept that immunodeficiency (downregulation of the immune system - the hand with thumb down) and autoimmunity (upregulation of the immune system - the hand with thumb up) appear different categories of human pathology, the former more related to genetics, the latter to environment and epigenetics, while the interplay between these two apparently distinct categories is guaranteed by the common line of the innate and acquired immunity, which is dysregulated in both.

It is paradoxical that patients with CVID have a high frequency of associated autoimmune features. Increasing pathogenetic insights allowed to reconcile the lack of B-cell maturation and autoimmunity in the wider concept of dysregulated immune system, both diseases being influenced by genetic and epigenetic factors which can lead to different clinical phenotypes (**Figure 1**).

The association of hypogammaglobulinemia and autoreactive B cells in CVID patients has been commonly listed as “SLE-like”. However, we propose that the autoimmunity and lymphoproliferation associated with CVID is more closely associated with a SS-like picture of immune dysregulation. In this context, CVID and SS, two conditions which can occur simultaneously and share several pathogenetic aspects, as well as targeted therapy (i.e., rituximab, abatacept), could represent a model of this immunological view. Therefore, better understanding of the underlying immunological mechanisms and specific genetic mutations that result in the immune dysregulation may lead to the development of new therapeutic targets for both the diseases.

## Author Contributions

LQ, SV, RF, and GM conceived the study. All authors contributed to the literature review and interpretation of the data. The first draft of the manuscript was written by LQ, RF, SV, GM, VM, SL, and MR, and all authors commented on previous versions of the manuscript. All authors contributed to the article and approved the submitted version.

## Conflict of Interest

The authors declare that the research was conducted in the absence of any commercial or financial relationships that could be construed as a potential conflict of interest.
